# miR-106a-5p Functions as a Tumor Suppressor by Targeting VEGFA in Renal Cell Carcinoma

**DOI:** 10.1155/2020/8837941

**Published:** 2020-11-08

**Authors:** Jun Ma, Wenguang Wang, Baihetiya Azhati, Yujie Wang, Hamulati Tusong

**Affiliations:** Department of Urology, The First Affiliated Hospital of Xinjiang Medical University, Urumqi 830054, China

## Abstract

MicroRNAs (miRNAs) regulate progression of different cancers. Nevertheless, there is limited information regarding the role of miR-106a-5p in renal cell carcinoma (RCC). Herein, we demonstrate that miR-106a-5p levels are drastically decreased in clear cell RCC (ccRCC) tissues and cell lines, which subsequently contribute to a poor patient overall survival and a high tumor stage. By screening and analyzing, we found that miR-106a-5p directly targets the 3′-UTR of the VEGFA mRNA and led to a decrease in VEGFA. This process is important for tumor cells' growth and colony formation, and overexpression of miR-106a-5p can especially kill kidney tumor cells. Therefore, our data reveal that miR-106a-5p functions as a tumor suppressor by regulating VEGFA and ccRCC may be susceptible to miR-106a-5p therapy.

## 1. Introduction

Renal cell carcinoma (RCC) predominates kidney cancers and encompasses many subtypes, of which clear cell RCC (ccRCC) is frequently diagnosed [1, 2]. Despite the development of surgical technology, its prognosis following metastasis or recurrence remains poor [3].

miRNAs, small noncoding RNAs, encompass 19 to 24 nucleotides and have vital roles in mRNA degradation and translational inhibition [4–7]. miRNAs function across a variety of cancers by regulating tumor growth, invasion, and metastasis [8–11]. Various functions of microRNA-106a-5p, part of the miR-17 family, via targeting different mRNA have been reported in many tumors [12–14], and miR-106a-5p levels are frequently lower in these tumors suggesting a tumor suppressor function of miR-106a-5p, especially in astrocytoma, osteosarcoma, and ovarian cancer [15–17]. However, little is known regarding miR-106a-5p in ccRCC.

Herein, we demonstrate that miR-106a-5p is substantially lower in ccRCC tissues in comparison to adjacent normal tissues from both our cohort and TCGA data. We further found that vascular endothelial growth factor A (VEGFA), part of the PDGF/VEGF growth factor family and key factors in tumor vessel formation [18], is a miR-106a-5p target at the 3′-UTR of the VEGFA mRNA and miR-106a-5p contributes to a decrease in VEGFA. VEGFA has been shown to have an important function in tumor growth, metastasis, and survival [19, 20]. High miR-106a-5p levels can decrease VEGFA levels and especially kill kidney tumor cells. Therefore, our data reveal a tumor suppressor role of miR-106a-5p by targeting VEGFA, and ccRCC may be susceptible to miR-106a-5p therapy.

## 2. Result

### 2.1. miR-106a-5p Levels Are Decreased in ccRCC and Correlate with the Outcomes of ccRCC Patients

**Figure 1:**
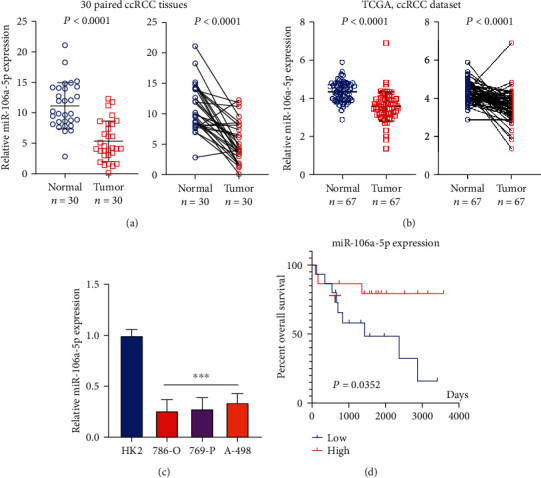
miR-106a-5p is downregulated in ccRCC and correlates with the outcomes of ccRCC patients. (a) miR-106a-5p levels across 30 ccRCC tissues in comparison to adjacent normal tissues were assessed utilizing RT-qPCR. (b) miR-106a-5p levels in patients' ccRCC tissues as per TCGA database. (c) miR-106a-5p levels across different cell lines. Each experiment was conducted three times. The data is indicated by means ± SD. (d) Overall survival Kaplan-Meier curves of miR-106a-5p in 30 ccRCC patients were plotted.

Studies suggest that miR-106a-5p is decreased in osteosarcoma, astrocytoma, and ovarian cancer [15–17]. Herein, we investigated the miR-106a-5p expression level in clear cell RCC (ccRCC). Overall, 30 ccRCC tissues and paired normal adjacent tissues from our cohort were collected for the detection of miR-106a-5p expression via qRT-PCR. We discovered that miR-106a-5p was substantially lower in tumor samples in comparison to adjacent normal tissues whether in a paired or unpaired group (Figure 1(a)). Additionally, we verified the miR-106a-5p levels from TCGA dataset [21], and the result is consistent (Figure 1(b)). We further investigated the miR-106a-5p expression in three human RCC cell lines (786-O, 769-P, and A-498), as well as normal renal tubular epithelial HK2 cells. Interestingly, miR-106a-5p was substantially increased in HK2 cells in comparison to RCC tumor cells (Figure 1(c)). Then, we suspect that this decrease in miR-106a-5p in tumors may be related to clinical outcomes, and the Kaplan-Meier analysis from our cohort indicated that a reduction of miR-106a-5p was correlated with reduced overall survival time of ccRCC patients (Figure 1(d)) and a higher Fuhrman grade (Table 1). Our finding indicates that miR-106a-5p may serve as a tumor suppressor in ccRCC.

### 2.2. Identifying a Potential Target of miR-106a-5p

**Figure 2:**
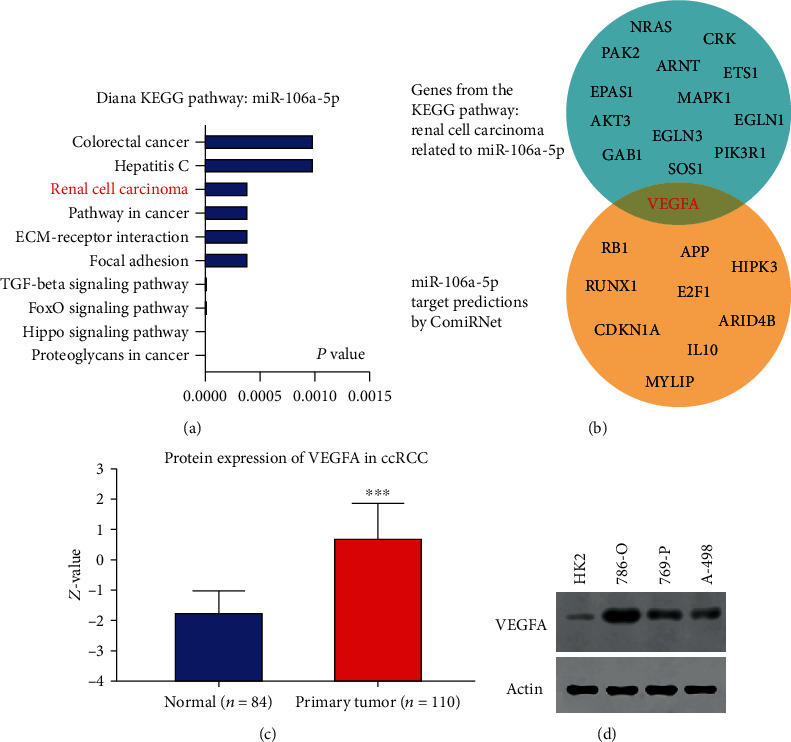
Identifying miR-106a-5p targets. (a) Diana KEGG pathway analysis of miR-106a-5p. (b) miR-106a-5p target was predicted by using ComiRNet and overlapped with the renal cell carcinoma gene set from (a). (c) Protein level of VEGFA in ccRCC patients from UALCAN. (d) Protein level of VEGFA in different cell lines.

MicroRNAs usually target special mRNA for degradation or translational inhibition [4–7]; how miR-106a-5p functions in ccRCC? We still barely know. Using Diana miRPath [22], we first analyzed the possible pathway correlated with miR-106a-5p and we found a special renal cell carcinoma gene set listed in the top ten related pathways of miR-106a-5p (Figure 2(a)). Then, we used ComiRNet [23], a web-based system, to assess the possible targets of miR-106a-5p, and we selected the top ten possible targets from ComiRNet and overlapped them with the renal cell carcinoma gene set from Diana miRPath; it happened when the VEGFA is the only overlapped gene (Figure 2(b)). Using the Clinical Proteomic Tumor Analysis Consortium (CPTAC) Confirmatory/Discovery dataset [24], we found that VEGFA protein in ccRCC patients' tumor tissues was substantially increased compared to that in normal tissues (Figure 2(c)). VEGFA protein levels are also higher in RCC cell lines in comparison to HK2 cells (Figure 2(d)). Since VEGFA has been reported functional in tumor growth and survival instead of only angiogenesis [19, 20], it might be an important target of miR-106a-5p for tumor suppression.

### 2.3. miR-106a-5p Directly Targets the 3′-UTR of VEGFA

**Figure 3:**
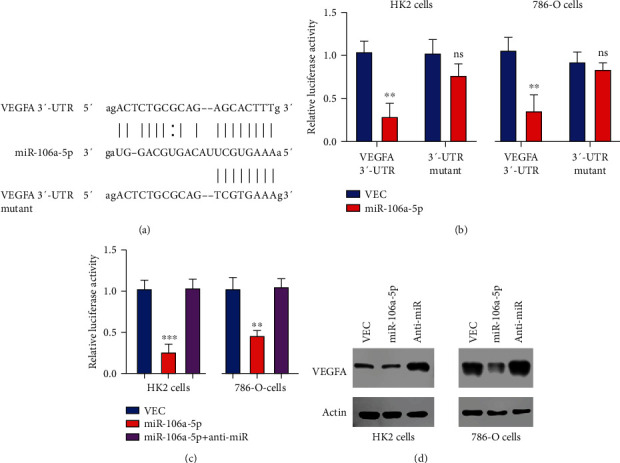
miR-106a-5p targets the 3′-UTR of VEGFA. (a) Predicted binding positions among VEGFA and miR-106a-5p and mutant sites on VEGFA. (b) The dual-luciferase reporter assay helped identify reporter activity in HK2 and 786-O cells transfected with the indicated microRNA mimics and wild-type or mutant VEGFA 3′-UTR. (c) Dual-luciferase reporter assay transfected with the indicated microRNA mimics. (d) Protein level from cell harvest from (c).

After target screening of miR-106a-5p, we found that VEGFA is a candidate target, and the possible binding site from ComiRNet is shown in Figure 3(a). Next, we utilized a dual-luciferase assay to further confirm the predicted binding positions of VEGFA by miR-106a-5p. Both the wild-type and mutant VEGFA 3′-untranslated region (3′-UTR) constructs encompassing the predicted miR-106a-5p binding site were subcloned to a pMIR reporter plasmid. Luciferase activity was reduced after overexpression of miR-106a-5p in both HK2 and 786-O cells which were transfected with wild-type 3′-UTR of VEGFA, but not mutant 3′-UTR (Figure 3(b)). However, when cells were treated with an oligonucleotide inhibitor of miR-106a-5p, the luciferase activity of VEGFA 3′-UTR in both HK2 and 786-O cells was restored (Figure 3(c)). miR-106a-5p overexpression also caused a significant reduction of VEGFA protein level, which is reversed by its oligonucleotide inhibitor in both HK2 and 786-O cells (Figure 3(d)). This data further confirms that miR-106a-5p targets VEGFA.

### 2.4. miR-106a-5p Specially Kills Kidney Tumor Cells

**Figure 4:**
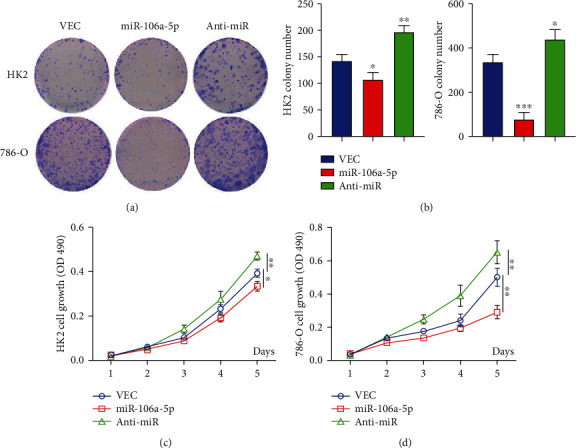
miR-106a-5p specially kills kidney tumor cells. (a, b) Colony formation assay from HK2 and 786-O cells transfected with the indicated microRNA mimics. The quantity of colonies was calculated. Representative colonies are shown in (a) with quantification data shown in (b). (c, d) Cell proliferation from (c) HK2 and (d) 786-O cells which were transfected with microRNA mimics. Data is represented as mean ± SD (*n* = 3). ^∗^*P* < 0.05, ^∗∗^*P* < 0.01, and ^∗∗∗^*P* < 0.001.

VEGFA plays important roles not just in angiogenesis but in tumor growth, metastasis, and survival as well [25–30]; that is why VEGFA is upregulated in tumors and necessary for tumor survival [31, 32]. Our finding revealed that miR-106a-5p can decrease VEGFA protein expression significantly; thus, we try to find out whether miR-106a-5p can specially kills kidney tumor cells. Colony formation assays demonstrate that miR-106a-5p overexpression can decrease the colony numbers partially in HK2 cells and but dramatically in 786-O cells; however, the oligonucleotide inhibitor of miR-106a-5p partially augmented the colony numbers significantly in HK2 cells but less in 786-O cells (Figures 4(a) and 4(b)). Using the cell proliferation assay, the results are consistent with the colony formation, miR-106a-5p decreased cell proliferation in 786-O cells but its inhibitor increased the proliferation, and less effect was found in HK2 cells (Figures 4(c) and 4(d)). Therefore, our finding demonstrates that miR-106a-5p specially kills tumor cells.

## 3. Discussion

miRNAs, small noncoding RNAs, are composed of 19 to 24 nucleotides and have vital functions in mRNA degradation and translational inhibition [33]. It is essential to explore the various functions of different miRNAs in different cellular and tumor context [34, 35]. A better understanding of miRNAs' targets and their functions in different diseases can benefit the therapy methods with these candidates, such as miRNA mimics or antimiRNA mimics [35, 36]. Our finding demonstrates that miR-106a-5p can represent a potential biomarker for ccRCC patients and increased miR-106a-5p levels can dramatically decrease the protein level of VEGFA in kidney tumor cells, consequently inhibiting tumor cell proliferation and leading to cell death. This data indicates that miR-106a-5p may be a candidate therapeutic target in ccRCC.

VEGFA is frequently upregulated in human cancers, and its important role in angiogenesis led to a development of therapies targeting VEGF and VEGF receptor [37], for example, bevacizumab, a monoclonal antibody that interrupts the interaction between VEGFA and its receptors (VEGFR1 and VEGFR2) and has already been used in a clinical setting [38]. However, a limited efficacy of VEGF-targeted therapeutics has been reported to have short responses in the majority of solid tumors [39]. The most common failure of these VEGF-targeted therapies is VEGFA upregulation, which then contributes to angiogenic therapy failure and disease recurrence [40]. Our finding demonstrates that miR-106a-5p can be a reason for VEGFA upregulation in tumor cells. Due to technical reasons, we did not perform assays related to angiogenesis, but overexpressing miR-106a-5p already should have a good effect on killing tumor cells, so we suspect that when the tumor microenvironment is involved, this tumor-killing ability of miR-106a-5p could be further improved.

Taken together, we discovered that miR-106a-5p is tightly related to ccRCC development and growth. miR-106a-5p directly targets the 3′-UTR of the VEGFA mRNA and contributes to a decrease in VEGFA. This process is important for tumor cells' growth and colony formation, and overexpression of miR-106a-5p can specially kill kidney tumor cells. Therefore, our data reveal that miR-106a-5p has a tumor suppressor role in RCC by targeting VEGFA and provides a possible treatment target for ccRCC.

## 4. Material and Methods

### 4.1. Patients and Tissue Specimens

Overall, 30 human ccRCC tissues, as well as adjacent normal tissues, were surgically collected through patients at the First Affiliated Hospital of Xinjiang Medical University from April 2013 to December 2016. Informed consent was provided by all patients, and the clinical study and analysis were granted approval by the Ethics Committee of the First Affiliated Hospital of Xinjiang Medical University (Urumqi, China).

### 4.2. Cell Culture

Human RCC cell lines (786-O, 769-P, and A-498) and the human renal tubular epithelial cell line (HK2) were kept in our lab and previously acquired through the cell bank (Shanghai, China). RPMI 1640 medium (HyClone, Logan, USA) was used to maintain 786-O, 769-P, and A-498 cells, which was supplemented with 10% (*v*/*v*) fetal bovine serum (FBS; Gibco, Shanghai, China). Dulbecco's modified Eagle's medium (HyClone) was used to grow HK2 cells with 10% (*v*/*v*) FBS. A condition of 37°C with 5% CO_2_ was used for culturing all cells.

### 4.3. RNA Purification and RT-PCR

TRIzol (Invitrogen; Thermo Fisher Scientific, Inc.) was utilized to isolate RNA through tissue samples and cells after purification with an RNeasy Maxi Kit (Qiagen, Inc., Santa Clarita, CA, USA). A TaqMan MicroRNA Reverse Transcription Kit (Thermo Fisher Scientific, Inc.) was utilized for reverse transcription. The LightCycler 480 Real-Time PCR system (Roche Diagnostics, Basel, Switzerland) and miScript SYBR Green PCR Kit (Qiagen) were used for the qPCR assay. TBP and U6 served as internal controls for VEGFA and miR-106a-5p, respectively.

### 4.4. Bioinformatics Methods

The predicted targets of miR-106a-5p were identified and analyzed through the use of ComiRNet (http://comirnet.di.uniba.it/) and Diana (http://diana.imis.athena-innovation.gr/). TCGA data was analyzed by cBioPortal (https://www.cbioportal.org/). The protein level of VEGFA in patients is obtained from UALCAN (http://ualcan.path.uab.edu/analysis-prot.html).

### 4.5. Oligonucleotide Transfection

Negative control mimics (mimics-NC), miR-106a-5p, and miR-106a-5p inhibitor were obtained through GENE-UP (Shenzhen, China). Lipofectamine 3000 (Invitrogen, USA) was utilized for transfection.

### 4.6. Luciferase Reporter Assay

Wild-type or mutant 3′-UTR of VEGFA mRNA was subcloned into a pMIR-REPORT luciferase vector (Applied Biosystems, USA). miR-106a-5p or negative control mimics with the reporter vector were transfected in cells 24 hours postseeding into 12-well plates through the Lipofectamine 2000 reagent (Invitrogen, USA). Luciferase activities were evaluated through the Dual-Luciferase Assay System (Promega, USA) after 48 hours.

### 4.7. Colony Formation Assay

100 cells were calculated and plated into 6-well plates after 24-hour transfection. Dilute crystal violet (1 : 30) and acetic acid and methanol (1 : 4) were used to fix cells after growing for two weeks. The colony number was counted manually. Each trial was conducted three times.

### 4.8. Cell Proliferation Assay

100 cells were calculated and placed into 96-well plates after miRNA transection. The OD value was quantified at 490 nm in a SpectraMax 190 spectrophotometer (Molecular Devices) after cells were grown for 2 h at 37°C and treated with methylthiazolyldiphenyl-tetrazolium bromide (MTT, 1 mM, Sigma).

### 4.9. Statistical Analysis

The Kaplan-Meier method was utilized for generating the overall survival (OS) curve. Each numerical data point is represented as mean ± SD. The variation between groups is compared using a *t*-test. ^∗^*P* < 0.05, ^∗∗^*P* < 0.01, and ^∗∗∗^*P* < 0.001.

## Figures and Tables

**Table 1:** Correlation between miR-106a-5p expression and clinicopathological variables in 30 ccRCC patients.

Characteristics	Total	miR-106a-5p expression		*P* value
		Low (*N* = 15)	High (*N* = 15)	
Gender				0.7125
Male	13	7	6	
Female	17	8	9	
Age				0.6903
<60 years	21	10	11	
≥60 years	9	5	4	
Tumor size				0.7048
<3 cm	19	9	10	
≥3 cm	11	6	5	
Fuhrman grade				0.02535
I-II	12	3	9	
III-IV	18	12	6	

## Data Availability

Source data and reagents are available from the corresponding author upon reasonable request.
